# Structures and functions of insect arylalkylamine *N*-acetyltransferase (iaaNAT); a key enzyme for physiological and behavioral switch in arthropods

**DOI:** 10.3389/fphys.2015.00113

**Published:** 2015-04-13

**Authors:** Susumu Hiragaki, Takeshi Suzuki, Ahmed A. M. Mohamed, Makio Takeda

**Affiliations:** ^1^Graduate School of Agricultural Science, Kobe UniversityKobe, Japan; ^2^Department of Biology, The University of Western OntarioLondon, ON, Canada

**Keywords:** serotonin (5HT), melatonin (MEL), arylalkylamine *N*-acetyl transferase (aaNAT), arylamine *N*-acetyltransferase, circadian rhythms, photoperiodism, UV adaptation

## Abstract

The evolution of *N*-acetyltransfeases (NATs) seems complex. Vertebrate arylalkylamine *N*-acetyltransferase (aaNAT) has been extensively studied since it leads to the synthesis of melatonin, a multifunctional neurohormone prevalent in photoreceptor cells, and is known as a chemical token of the night. Melatonin also serves as a scavenger for reactive oxygen species. This is also true with invertebrates. NAT therefore has distinct functional implications in circadian function, as timezymes (aaNAT), and also xenobiotic reactions (arylamine NAT or simply NAT). NATs belong to a broader enzyme group, the GCN5-related *N*-acetyltransferase superfamily. Due to low sequence homology and a seemingly fast rate of structural differentiation, the nomenclature for NATs can be confusing. The advent of bioinformatics, however, has helped to classify this group of enzymes; vertebrates have two distinct subgroups, the timezyme type and the xenobiotic type, which has a wider substrate range including imidazolamine, pharmacological drugs, environmental toxicants and even histone. Insect aaNAT (iaaNAT) form their own clade in the phylogeny, distinct from vertebrate aaNATs. Arthropods are unique, since the phylum has exoskeleton in which quinones derived from *N-*acetylated monoamines function in coupling chitin and arthropodins. Monoamine oxidase (MAO) activity is limited in insects, but NAT-mediated degradation prevails. However, unexpectedly iaaNAT occurs not only among arthropods but also among basal deuterostomia, and is therefore more apomorphic. Our analyses illustrate that iaaNATs has unique physiological roles but at the same time it plays a role in a timezyme function, at least in photoperiodism. Photoperiodism has been considered as a function of circadian system but the detailed molecular mechanism is not well understood. We propose a molecular hypothesis for photoperiodism in *Antheraea pernyi* based on the transcription regulation of *NAT* interlocked by the circadian system. Therefore, the enzyme plays both unique and universal roles in insects. The unique role of iaaNATs in physiological regulation urges the targeting of this system for integrated pest management (IPM). We indeed showed a successful example of chemical compound screening with reconstituted enzyme and further attempts seem promising.

## Historical aspects and reality in insect aaNAT

Arylalkylamine *N*-acetyltransferase (aaNAT) has been most extensively studied as a penultimate enzyme for melatonin (MEL) synthesis in vertebrates (Klein and Weller, 1970; Axelrod, 1974; Ebisawa and Deguchi, 1991; Chong et al., 2000; Iuvone et al., 2005; Falcón et al., 2014), particularly in the context of daily and seasonal timing for activity, metabolism and physiological phenomena, thus commonly called timezyme (Klein, 2007). This enzyme is expressed in the pineal gland, retina, and parietal eyes that are photosensitive organs but the exocrine Hardelian gland also contains MEL synthesizing machinery (Dieridane and Touitou, 2001). A massive amount of MEL has been reported in the gastroenteric tract in mammals (Bubenik, 2008). The number of genes for aaNAT is one in higher vertebrates (Sabbagh et al., 2013). However, multiple acetylases are known in vertebrates that function as xenobiotic enzyme, ex. seven in cephalochordate and three in human (Pavlicek et al., 2010; Sabbagh et al., 2013), and human subject has polymorphism in this gene to symptomatically separate forms commonly termed slow and fast-acetylators. This enzyme has been called arylamine *N*-acetyltransferase (Ebisawa and Deguchi, 1991; Deguchi, 1992).

In arthropods especially insects, more extensive use of this enzyme is made for irrelevant functions to vertebrates such as sclerotization where dopamine, tyramine, norepinephrine and octopamine are *N-*acetylated to provide reactive quinones for linking chitin and arthropodins forming a cuticular matrix (Karlson et al., 1962; Sekeris and Karlson, 1962). The enzyme was first called dopamine acetyltransferase (DAT). Also, metabolic pathways of neurotransmitter monoamines are different from vertebrates; in vertebrates monoamines are metabolized mainly by monoamine oxidases (MAO1 and 2) but in arthropods the activity of MAOs is usually limited and these amines are metabolized mainly by NAT instead (Dewhurst et al., 1972; Sloley, 2004), though oxidized products are substantially detected during diapause in *Antheraea pernyi* at the pupal stage (Matsumoto and Takeda, 2002).

Since major neurotransmitter metabolism depends on this enzyme, it serves as an important regulator in a variety of physiological functions, metabolism, developmental determination, reproduction and behavior. Activities of aaNAT have been observed in various organs not only the CNS but also in the midgut, reproductive glands of both sexes and Malpigian tublules in the cockroach, *Periplaneta americana*, for example (Ichihara et al., 1997, 2001; Asano and Takeda, 1998; Asano et al., 2003). Isolation and characterization of aaNAT revealed that the activities are separated to different fractions, each showing a unique optimal pH and kinetic character (Ichihara et al., 1997, 1998, 2001; Asano and Takeda, 1998; Sakamoto et al., 1998), suggesting that aaNAT activities consist of a family of enzymes. Two isoforms have been isolated from testicular accessory gland and midgut. Both forms had 28 kDa molecular mass in SDS-PAGE but the former had an optimal pH at 5–6 whereas the latter 9–10 and thus called acidic aaNAT (aNAT) and basic aaNAT (bNAT), respectively (Ichihara et al., 1997, 2001; Bembenek et al., 2005a,b). V_max_ and K_m_-values estimated from a purified fraction of both forms are listed in Table 1.

**Table 1:** **Comparison in kinetic properties of two forms of isolated aaNAT from *P. americana*. aNAT/bNAT**.

**Substrate**	**Km (mM)**	**Vmax (μmol/min/mg protein)**
Tryptamine	0.025/1.33	11.2/276
5HT	0.05/0.325	9.33/110
Dopamine	0.025/1.52	6.22/172
Octopamine	0.02/0.385	9.33/276
Tyramine	0.0286/0.0157	18.7/420
Methoxy-TN	0.025/0.2	6.57/230
Norepinephrine	0.0286/-	12.4/-

In *Drosophila melanogaster*, two aaNAT isoforms have been purified and aaNAT1 has been detected by *in situ* hybridization in the brain, ventral cord, midgut and oenocyte (Hintermann et al., 1996). aaNAT2 had 30% identity to aaNAT1 and estimated molecular mass was 24.4 vs. 31.0 kDa of NAT1 and IEP, 5.02 vs. 5.08 (Amherd et al., 2000). MEL rhythm had bimodal peaks but both enzymes failed to show circadian fluctuation in transcript amount (Hintermann et al., 1996; Amherd et al., 2000), while aaNAT of cockroach and *A. pernyi* showed a circadian rhythm in activity and in transcription (Bembenek et al., 2005a,b; Mohamed et al., 2014).

## Kinetic aspects: how many enzymes?

There was a practical reason for the difficulty to isolate aaNAT in vertebrates. This enzyme is extremely sensitive to light in vertebrates even at second order. Therefore, the detection required Bolton-Hunter labeling, apparently too small an amount for sequence determination (Namboodiri et al., 1987). However, differential cloning successfully identified aaNAT before purification was completed (Borjigin et al., 1995; Coon et al., 1995). However, iaaNAT was much more photorefractory and HPLC resulted in a purified fraction. The protein sequence was determined in cleavage products from cDNA cloned from the accessory gland of male gonad of *P. americana* (Ichihara et al., 1997, 1998; Bembenek et al., 2005b). Chromatographic isolation showed that at least two types of NATs occurred at different fractions that can be characterized by pH optima and substrate specificity. We tentatively called the two types, NATa and b that had pH optima at acidic and basic side, respectively. Different organs contain different ratios of the mixture (Ichihara et al., 1997, 2001; Bembenek et al., 2005b). The two types show different affinity to serotonin (5HT)/tryptamine and octopamine. The combination of amine substrate and pH using samples isolated from the accessory gland of cockroach at different ages showed at least four distinct patterns of change along ovulation cycle (Asano et al., 2003). Since homology of amino acid sequence was low among aaNATs, the ordinary PCR approach was not successful, but functional domains were relatively conserved and from the *Bombyx* EST database, cDNA was amplified from *Bombyx mori* brain-subesophageal ganglion (SOG) (Tsugehara et al., 2007). The cDNA encodes a 261 amino acid protein and Baculovirus-mediated reconstituted enzyme had a wide range of substrate affinity including tryptamine, 5HT, dopamine, octopamine and norepinephrine (Tsugehara et al., 2007). The transcript was expressed in different tissues such as head, ovary, testes and even flight muscles and in eggs, larvae, and adults. It showed a substrate inhibition by a high concentration of tryptamine. We also obtained cDNA for aaNAT from *A. pernyi* (Tsugehara et al., 2013), expressing this enzyme in Sf9 cell line similarly and confirmed its enzymatic activity (Tsugehara et al., 2013). It had the optimal pH at 8.0 and apparent K_m_ and V_max_ 1.42 μM and 154 nmol/min/mg protein with acetyl CoA and 3.31 μM and 190 nmol/min/mg with tryptamine. Substrate inhibition was observed above 0.03 mM with 0.11 mM acetyl CoA. Since the enzyme employs two substrates, arylalkylamines and acetyl CoA, we made a bisubstrate kinetic analysis employing brain-SOG and collaterial gland homogenates of *P. americana*. The former followed ping pong Bi-Bi mechanism and the latter sequential Bi-Bi mechanism, respectively (Asano and Takeda, 1998; Sakamoto et al., 1998).

## Functions of aaNAT in vertebrates

aaNAT transfers acetyl moiety to 5HT and controls daily changes in circulating MEL in boney vertebrates (Ganguly et al., 2002). In mammals, only the suprachiasmatic nucleus (SCN) serves as a circadian pacemaker (CPM) that receives light signals from the retina, and directly regulates pineal MEL biosynthesis as an output of the clock (Bell-Pedersen et al., 2005). The rhythmic pattern of activity in the MEL pathway is conserved among vertebrates, consistent with the role of MEL as the vertebrate hormone of time, i.e., high level signal at night and low level signal day. MEL is involved in numerous physiological processes including circadian entrainment, blood pressure regulation, oncogenesis, retinal physiology, ovarian physiology, and immune function (Altun and Ugur-Altun, 2007). As another notable example, cycling expression of the clock gene *Period1* in rodent pars tuberalis (PT) completely depends on the nocturnal activation of MEL receptor type-1 (MT1; von Gall et al., 2002). Cyclic *Period1* expression in PT is noteworthy because PT is where MEL acts as a mediator between CPM and output of photoperiodism (Hoffman and Reiter, 1965; Messager et al., 1999; Masumoto et al., 2010). The remarkable role that aaNAT plays in vertebrate time keeping monikered the aaNAT as “the timezyme” (Klein, 2007).

The aaNAT family, belonging to the GCN5 acetyltransferases (GNAT) superfamily (Dyda et al., 2000) is classified as vertebrate (VT) aaNAT and non-vertebrate (NV) aaNAT based on sequence similarity. Striking features of VT-aaNAT are found in regulatory and catalytic regions of the encoded proteins (Ganguly et al., 2001; Coon and Klein, 2006; Pavlicek et al., 2010). VT-aaNAT exists exclusively in two organs, the pineal gland and retina, both photosensitive (Coon et al., 1995). NV-aaNAT performs a detoxification function through acetylation of a broad range of arylalkylamines and polyamines in throughout body segment (Ganguly et al., 2001; Pavlicek et al., 2010), in contrast to VT-aaNAT which plays as a timezyme (Klein, 2007).

According to similarity to VT-aaNAT and NV-aaNAT, these aaNATs have been found in Gram-positive bacteria, fungi, some lower plants, algae, Placozoa, Annelida, Cephalochordates, and Vertebrates (Coon and Klein, 2006; Falcón et al., 2014) but not in other published genomes, including higher plants, nematodes and arthropods (Coon and Klein, 2006). In addition, both VT- and NV-aaNATs are not found in Echinodermata, Hemichordates, and Urochordates, which leaves an open question of when and how NV-aaNAT first appeared in Deuterostomia evolution (Pavlicek et al., 2010). Iyer et al. (2004) have developed a hypothesis that a horizontal gene transfer from bacteria to an ancestor of vertebrate caused the evolution of VT-aaNAT. The homologs in amphioxus, unicellular green algae, fungi and bacteria lack regulatory sequences found in VT-aaNAT. Also, all these are intronless. These features are consistent with a horizontal gene transfer of the aaNAT ancestor from bacteria to green algae, fungi and Cephalochordates (Coon and Klein, 2006). Very recently, two types of aaNAT were cloned from Chondrichthyes (Falcón et al., 2014). One was found in the pineal gland and retina, structurally, biochemically, and kinetically similar to VT-aaNAT. The other occurs throughout the body like NV-aaNAT. Calculation of the evolutionary rates revealed an acceleration of evolution by approximately an order of magnitude in the stem of the VT-aaNAT subfamily (Falcón et al., 2014). Therefore, the emergence of VT-aaNAT timezyme apparently dramatically accelerated evolution, accompanied with neofunctionalization after the NV-aaNAT gene duplication.

## Function of insect-type aaNAT

aaNATs play multiple roles in behavioral and physiological regulations in insects. Insect aaNAT (iaaNAT) is involved in cuticular sclerotization and suppression of black pigmentation (Dai et al., 2010; Zhan et al., 2010; Osanai-Futahashi et al., 2012). iaaNAT inactivates biogenic amines, such as octopamine, dopamine, and 5HT (Hintermann et al., 1995; Tsugehara et al., 2007), since there is little or no MAO activity within the insect nervous tissue (Sloley, 2004) unlike mammals (Bortolato et al., 2008). Following evidence indicates that iaaNATs regulate physiological events via MEL in insects; orally administrated MEL synchronizes circadian locomotor rhythms of the house cricket *Acheta domesticus* (Yamano et al., 2001) and MEL stimulates the release of a prothoracicotropic hormone (PTTH) in the brain of *P. americana* (Richter et al., 2000).

Very recently, we demonstrated that iaaNAT is the critical joint between the circadian system and photoperiodism in *A. pernyi* (Mohamed et al., 2014). It is noteworthy that the function of iaaNAT has several striking similarities to that of VT-aaNAT in the endocrine switch, as it is supported by the following evidence. (1) *iaanat* is highly likely controlled by a negative feedback loop via E-box element. In the *iaanat* promoter region (1596 bp upstream region), there were 2 perfect E-boxes (CACGTG), 4 canonical E-boxes (CANNTG). qRT-PCR revealed rhythmical expression of *iaanat* in LD cycle. RNAi against *per*, putative negative regulator of E-box, resulted in up-regulation of *nat* transcription. In contrast, RNAi knockdown of *Clk* and *cyc*, positive regulator of E-box, suppressed the expression level of *nat*. In chicken, the abundance of VT-aaNAT mRNA in the pineal gland exhibits a circadian rhythm (Bernard et al., 1997), which is regulated by BMAL1/CLOCK and BMAL1/MOP4 heterodimers via E-box (Chong et al., 2000). (2) iaaNAT regulates photoperiodism. dsRNA^iaaNAT^-injected pupae failed to emerge even under long day, whereas pupae injected with dsRNA^per^ showed a high proportion of emergence even under short day. In mammals, the nocturnal MEL secretion provides an endocrine signal of photoperiod to the PT to regulate thyroid-stimulating hormone which regulates seasonal reproduction (Yoshimura, 2013). (3) MEL content is regulated by the expression level of *iaanat*. Injection of dsRNA^iaaNAT^ induced a great decline of MEL in 48 h. In contrast, MEL level was significantly higher than control pupae after knocking down of *per* by 24 h. In higher vertebrates, the switch between day and night profiles of pineal MEL is driven by changes in the activity and expression level of VT-aaNAT, which increases at night 10- to 100-fold (Ganguly et al., 2002). (4) iaaNAT located at a unique from neuroendocrine cells which control photoperiodisum. iaaNAT-like immunoreactivity (-ir) was observed in the PER/CYC/CLK-ir cells, putative “clock neurons.” MT2-ir and PTTH-ir were co-localized in a pair of neurosecretory neurons juxtaposing the putative clock neurons. In mammals, VT-aaNAT is highly expressed in the pineal gland, which is near MT1-expressing tissue, PT (Nakane and Yoshimura, 2014).

According to the significance and similarity to the original timezyme, we considered that iaaNAT in *A. pernyi* serves as a switch of PTTH secretion despite no universally accepted theory for the function of iaaNAT as timezyme to date in insects since iaaNAT family has primary structure distinct from VT-aaNAT and NV-aaNAT. These families are only distantly related to each other, although both belong to the GNAT superfamily (Falcón et al., 2014).

## Phylogenetic relationship of aaNATs depicted from metazoan genome study

Insects do not have VT-aaNAT or NV-aaNAT homologs, based on sequence similarity searches of public genomic sequences (Pavlicek et al., 2010); however, insects do have multiple aaNAT enzymes that show very low sequence identity with VT-aaNAT/NV-aaNAT. According to enzymatic properties of aaNAT, insects seems to have more than 4 aaNAT-like enzymes in a single species as described before. The biochemical characterizations of iaaNATs were followed up with molecular isolation in *D. melanogaster* (Hintermann et al., 1995, 1996; Amherd et al., 2000), *B. mori* (Tsugehara et al., 2007), *A. pernyi* (Tsugehara, 2006), and *P. americana* (Ichihara et al., 1997, 2001; Bembenek et al., 2005a,b). Primary structure of iaaNATs show very low sequence identity between each other (Hintermann et al., 1996; Amherd et al., 2000; Bembenek et al., 2005b; Tsugehara et al., 2007). How many iaaNATs exist in one species? How is the relationship between each iaaNAT? To answer these questions, exhaustive analysis of iaaNAT is required.

A first remarkable genome-wide characterization of iaaNATs was held on several dipteran species, *Anopheles gambiae, Aedes aegypti, Culex quinquefasciatus*, and *D. melanogaster* (Mehere et al., 2011; Han et al., 2012). They studied three proteins in detail: iaaNAT2, iaaNAT5b, and putative iaaNAT7 (piaaNAT7) from *A. aegypti*, each from a different cluster of phylogenetic trees. All three iaaNAT and sheep VT-aaNAT structures have a common fold core of GNAT superfamily proteins, along with a unique structural feature: helix/helices between β3 and β4 strands.

Subsequently, genome-wide characterization of iaaNATs was held on the pea aphid, *Acyrthosiphon pisum* (Barberà et al., 2013). They yielded five predicted sequences with *E*-values lower than 1.0 × 10^−4^ in BLASTP search on the *A. pisum* genomic database using *D. melanogaster* (*Dm*) DAT as a query. Following screening using conserved domain (CD)-search tool available at the National Center for Biotechnology Information identified four iaaNATs (iaaNAT1, iaaNAT2, iaaNAT3, and iaaNAT4). Among them, two (iaaNAT1 and iaaNAT3) showed highly significant variation in transcription levels depending on photoperiods.

**Figure 1:**
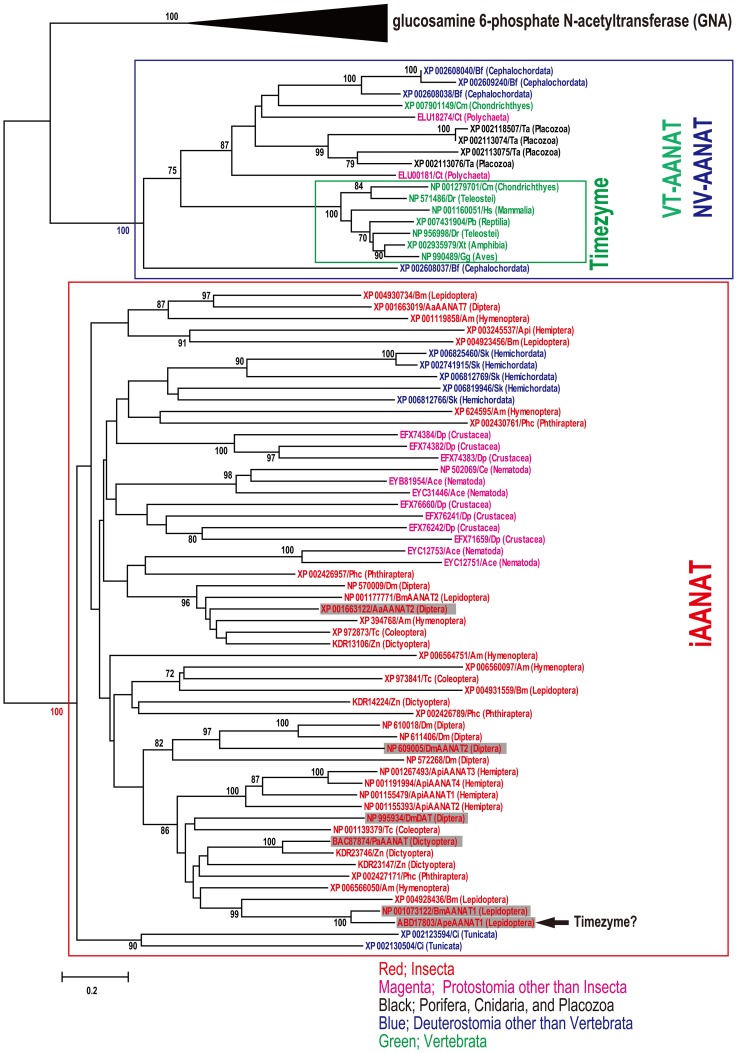
**Neighbor-joining tree built using Poisson-corrected distances on different arylalkylamine *N*-acetyltransferases (aaNATs) including insect aaNATs, vertebrate aaNATs, and non-vertebrate aaNATs (iaaNATs, VT-aaNATs, and NV-aaNATs, respectively)**. Glucosamine 6-phosphate *N*-acetyltransferases (GNAs) were used as outgroups. The bootstrap values of 500 replicates are shown as percentages at nodes. Bootstrap values are only shown for nodes with greater than 70% support. The clades in red, blue and green squares indicate iaaNATs, NV-aaNATs/VT-aaNATs, and VT-aaNATs, respectively. Accession No., species abbreviation, and taxonomic information written in red, magenta, blue, and green indicate insecta, protostomia other than insecta, deuterostomia other than vertebrata, and vertebrata, respectively. Enzymatically characterized iaaNAT is indicated in shade. Arrow shows potential iaaNAT possess a function as a timezyme (Mohamed et al., 2014). Species abbreviations; *Dm, Drosophila melanogaster*; *Aa, Aedes aegypti*; *Am, Apis mellifera, Bm, Bombyx mori*; *Ape, Antheraea pernyi*; *Tc, Tribolium castaneum*; *Api, Acyrthosiphon pisum*; *Zn, Zootermopsis nevadensis*; *Pa, Periplaneta americana*; *Phc, Pediculus humanus corporis*; *Dp, Daphnia pulex*; *Ce, Caenorhabditis elegans*; *Ace, Ancylostoma ceylanicum*; *Ct, Capitella teleta*; *Ta, Trichoplax adhaerens*; *Sk, Saccoglossus kowalevskii*; *Ci, Ciona intestinalis*; *Bf, Branchiostoma floridae*; *Cm, Callorhinchus milii*; *Dr, Danio rerio*; *Xt, Xenopus tropicalis*; *Gg, Gallus gallus*; *Pb, Python bivittatus*; *Hs, Homo sapiens*.

**Figure 2:**
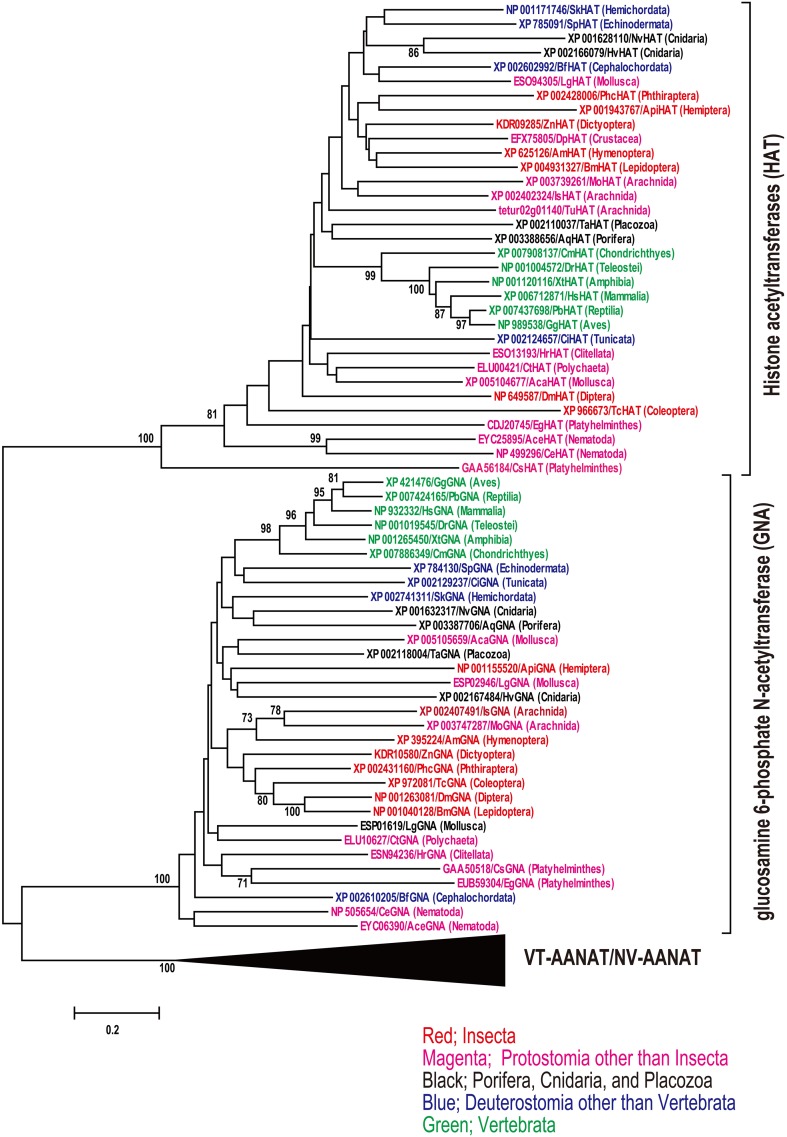
**Neighbor-joining tree built using Poisson-corrected distances on glucosamine 6-phosphate *N*-acetyltransferases (GNAs) and histone acetyltransferases (HATs) from same genomic databases used Figure 1 to check integrity of the genomic data**. Vertebrate aaNATs and non-vertebrate aaNATs (VT-aaNATs and NV-aaNATs, respectively) were used for outgroups. The bootstrap values of 500 replicates are shown as percentages at nodes. Bootstrap values are only shown for nodes with greater than 70% support. Accession No., species abbreviation, and taxonomic information written in red, magenta, blue, and green indicate insecta, protostomia other than insecta, deuterostomia other than vertebrata, and vertebrata, respectively. Species abbreviation; *Dm, Drosophila melanogaster*; *Am, Apis mellifera, Bm, Bombyx mori*; *Tc, Tribolium castaneum*; *Api, Acyrthosiphon pisum*; *Zn, Zootermopsis nevadensis*; *Phc*,*Pediculus humanus corporis*; *Dp, Daphnia pulex*; *Is, Ixodes scapularis*; *Mo, Metaseiulus occidentalis*; *Tu, Tetranychus urticae*; *Ce, Caenorhabditis elegans*; *Ace, Ancylostoma ceylanicum*; *Aca, Aplysia californica*; *Lg, Lottia gigantea*; *Ct, Capitella teleta*; *Hr, Helobdella robusta*; *Cs, Clonorchis sinensis*; *Eg, Echinococcus granulosus*; *Nv, Nematostella vectensis*; *Hv, Hydra vulgaris*; *Aq, Amphimedon queenslandica*; *Ta, Trichoplax adhaerens*; *Sp, Strongylocentrotus purpuratus*; *Sk, Saccoglossus kowalevskii*; *Ci, Ciona intestinalis*; *Bf, Branchiostoma floridae*; *Cm, Callorhinchus milii*; *Dr, Danio rerio*; *Xt, Xenopus tropicalis*; *Gg, Gallus gallus*; *Pb, Python bivittatus*; *Hs, Homo sapiens*.

According to a series of genome-wide identification of iaaNAT, we try comprehensive phylogenetic analysis of VT-aaNAT, NV-aaNAT, and iaaNAT among metazoan species for better understanding of potential functions of iaaNAT. Combination of similarity search and CD-search revealed 57 iaaNATs, 11 NV-aaNATs, and 7 VT-aaNATs from the public genome database described above (Table S1). To confirm the orthology of the iaaNATs, NV-aaNATs, and VT-aaNATs genes screened, we carried out a phylogenetic analysis that included all 75 sequences and glucosamine 6-phosphate *N*-acetyltransferases (GNAs) from the public genome database described above (Figure 1). VT-aaNAT/NV-aaNAT and iaaNAT genes clearly clustered into distinct groups (mean pairwise value 100%). As expected, only iaaNATs were isolated from insect species among different orders. In addition to insect, iaaNATs were detected from *Daphnia pulex* (Crustacea), *Caenorhabditis elegans* (Nematoda), and *Ancylostoma ceylanicum* (Nematoda) from protostome spices. As described previously, *Capitella teleta* (Annelida; Polychaeta), *Trichoplax adhaerens* (Placozoa), and *Branchiostoma floridae* (Cephalochordata) have NV-aaNATs, whereas most vertebrates have only VT-aaNATs except *Callorhinchus milii* (Chondrichthyes) that had both NV-aaNAT and VT-aaNAT (Coon and Klein, 2006; Falcón et al., 2014). Surprisingly, 4 iaaNATs and 2 iaaNATs were retrieved from genomic database of deuterostome species, *Saccoglossus kowalevskii* (Hemichordata) and *Ciona intestinalis* (Urochordata), respectively. Neither iaaNAT, VT-aaNAT, nor NV-aaNAT have been detected in *Ixodes scapularis* (Arachnida), *Metaseiulus occidentalis* (Arachnida), *Tetranychus urticae* (Arachnida), *Aplysia californica* (Mollusca), *Lottia gigantea* (Mollusca), *Helobdella robusta* (Annelida; Clitellata), *Clonorchis sinensis* (Platyhelminthes), *Echinococcus granulosus* (Platyhelminthes), *Hydra vulgaris* (Cnidaria), *Nematostella vectensis* (Cnidaria), *Amphimedon queenslandica* (Porifera), and *Strongylocentrotus purpuratus* (Echinodermata). To check integrity of the genomic data lacking known aaNAT, sequence similarity searches was conducted using other acetyltransferases belonging to GNAT superfamily, *Hs*GNA (NP_932332) and *Hs* histone acetyltransferase (HAT; XP_006712871) as queries. A phylogenetic analysis was carried out in all isolated GNAs, HATs, and VT-aaNAT/NV-aaNAT to confirm the orthology (Figure 2). Expectedly, GNAs, HATs, and VT-aaNATs/NV-aaNATs cluster into three groups, all strongly supported (mean pairwise value 100%) by neighbor-joining algorithm on Poisson-corrected distances. This strongly suggests unique distributions of aaNATs within phyla unlike other GNAT superfamily.

**Figure 3:**
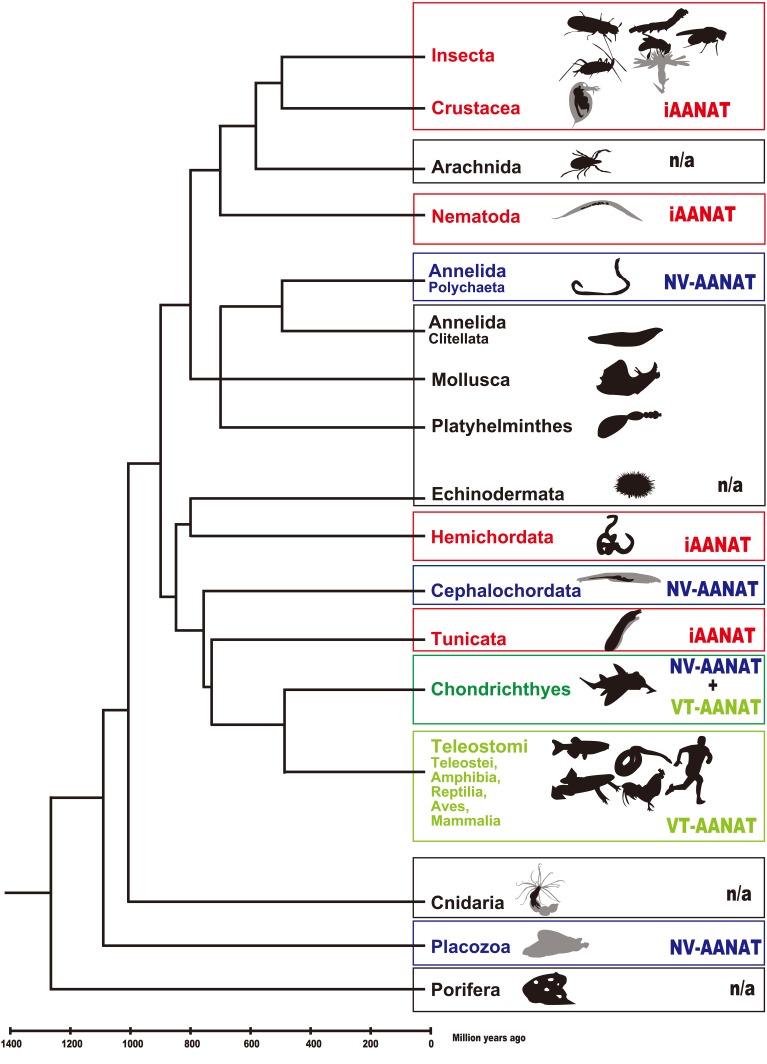
**Schematic phylogenetic time tree showing the phyla used in this study and divergence time**. The phylogenetic positions of tunicata, cephalochordata, and vertebrata are according to the results of molecular phylogenetic analysis of 1090 orthologous genes from whole genomic sequences (Putnam et al., 2008). Similarly, the phylogenetic position of placozoans relative to other metazoans is according to the results of Bayesian, maximum likelihood, and parsimony analyses concatenation of 104 slowly evolving singlecopy nuclear genes from fully sequenced genomes (Srivastava et al., 2008). The phyla in red, blue and light green squares indicate possession of iaaNATs, NV-aaNATs, and VT-aaNAT(s), respectively. The phyla in green square indicate possession of both NV-aaNATs and VT-aaNATs (Falcón et al., 2014). There are no phyla which have both iaaNAT and NV-aaNAT/VT-aaNAT among metazoan species so far.

The distribution of timezyme, VT-aaNAT and its ancestral form, NV-aaNAT is understood by gene duplications, deletion, and a horizontal gene transfer (Coon and Klein, 2006), followed by VT-aaNAT evolution from the NV-aaNAT associated with drastic neofunctionalization of aaNAT in the Cephalochordate-Vertebrate split (Falcón et al., 2014). However, according to diversity of aaNATs and current view of phylogenetic relationship of Metazoa (Figure 3), the evolution of aaNAT looks much more complex than expected. Controversial phylogenetic positions of Urochordata, Cephalochordata, and Vertebrata were accessed by Bayesian and maximum likelihood methods on a 1090 orthologous genes (Putnam et al., 2008). Both molecular phylogenetic analyses support that Cephalochordata represents the most basal extant chordate lineage in relation to Urochordata and Vertebrate. Also, the relationship of Placozoa to other animals was analyzed using Bayesian, maximum likelihood, and parsimony analyses of a concatenation of 104 slowly evolving single-copy nuclear genes (Srivastava et al., 2008). With 100% Bayesian support and 92% likelihood bootstrap support that Porifera diverged before the Placozoa-Cnidaria-Bilaterian clade. We found iaaNAT in Deuterostomia, such as Urochordata, a sister group between the Cephalochordate and Vertebrate. There are NV-aaNATs in Placozoa and Polychaeta but no NV-aaNATs in closely related species. iaaNATs are distributed in known genomic databases of Ecdysozoa but not in Arachnida in spite of distinct activity of aaNAT (Suzuki et al., 2008). No phyla have both iaaNAT and NV-aaNAT/VT-aaNAT among metazoan species so far. It is very difficult to explain in distribution of aaNATs if iaaNAT and NV-aaNAT/VT-aaNAT are not related at all. Comprehensive analyses of aaNATs are required.

## Spectral responses and non-visual photoreceptors

The activity of aaNAT shows a nocturnal increase and this enhances the biosynthesis of MEL at night (Baler et al., 1997). Deguchi and Axelrod (1972a) first developed a radioenzymatic measurement of aaNAT activity with [^14^C]-acetyl CoA. This highly-sensitive method clearly showed that aaNAT activity in the rat pineal organ increased 50-fold at night (Deguchi and Axelrod, 1972b). According to Korf (1994), aaNAT activity and subsequent secretion of MEL are suppressed by light signals from photoreceptors through endogenous oscillators, i.e., circadian clock. In mammals, the locations of photoreception, endogenous oscillation, and MEL secretion are the retina, SCN, and pineal organ, respectively. In contrast, pineal organ equips these three components in non-mammalian vertebrates (Falcón, 1999). First, researchers focused on the non-visual photoreceptors expressed in the pineal organs in non-mammalian vertebrates. Deguchi (1981) revealed that the isolated chicken pineal is sensitive to light (<700 nm) and that green light (λ_max_500 nm) is the most effective in suppressing the aaNAT activity. From the action spectrum, a rhodopsin-like molecule was suggested to function as photoreceptors in the pineal organ. However, the isolated photoreceptor was a blue sensitive pigment “pinopsin” (λ_max_468 nm) which is specifically expressed only in the pineal organ (Okano et al., 1994). Since the red-sensitive pigment “iodopsin” (λ_max_571 nm) was isolated from chicken pineal (Okano et al., 1989), cooperative photoreception by pinopsin and iodopsin is considered to suppress the aaNAT activity and results in the green-sensitive broad action spectrum (Deguchi, 1981). In addition, Blackshaw and Snyder (1997) identified an ultraviolet (UV)-sensitive pigment “parapinopsin” (λ_max_370 nm) in the catfish pineal and it was also isolated from the pineal organ of lampreys, fishes, and frogs (Koyanagi et al., 2004; Kawano-Yamashita et al., 2007). Furthermore, another opsin-based non-visual photopigment “melanopsin” (λ_max_480 nm) (Qiu et al., 2005) was identified from the photosensitive dermal melanophores of *X. laevis* (Provencio et al., 1998) and the homologs have been identified in varied vertebrates (Koyanagi and Terakita, 2008). In mammals, melanopsin is localized in retinal ganglion cells (RGCs) which project to SCN and suggested as a photoreceptor that sets the circadian clock (Berson et al., 2002; Hattar et al., 2002). Since blue light (446–477 nm) is most effective in suppressing human MEL (Brainard et al., 2001), light signals relayed from melanopsin expressed in the intrinsically photosensitive RGCs (ipRGCs) may suppress aaNAT activity in mammals. Interestingly, melanopsin analysis in the cephalochordate, the closest living invertebrate to the vertebrates showed an evolutionary link between vertebrate ipRGCs and invertebrate visual cells (Koyanagi et al., 2005). Similarities in structure, phylogenic position, and photochemical properties between vertebrate melanopsin expressed in ipRGCs and invertebrate visual pigments (opsin-based photopigments) expressed in rhabdomeric photoreceptor cells suggest a scenario that both ipRGCs and rhabdomeric photoreceptor cells are evolved from an ancestral photoreceptor cell and the former cells lost visual function (Provencio et al., 1998; Koyanagi et al., 2005). Therefore, visual pigments emerge as a strong candidate that mediates light-induced suppression of aaNAT activity in invertebrates as melanopsin in vertebrates (Foster and Hankins, 2007; Hankins et al., 2008).

In several invertebrates, spectral responses of aaNAT were recently reported. In *C. elegans*, blue light (450-500 nm) is the most effective in suppressing aaNAT activity (Migliori et al., 2012). In the band-legged cricket *Dianemobius nigrofasciatus*, the action spectrum for light-induced suppression of aaNAT activity also shows a highest peak at a blue light region (λ_max_450 nm) (Izawa et al., 2009). In the mite *T. urticae*, the main and second maxima of the action spectrum are at UV-A (350 nm) and blue light (450 nm) regions, respectively (Suzuki et al., 2009a). These spectral responses suggest that short-wave photopigments play a role as the input system for phototransduction for aaNAT regulation in invertebrates. From the spectral characteristics, not only opsin-based photopigments but also flavin-based photopigment “cryptochrome” which is a short-wave photopigment and functions as a photoreceptor for circadian entrainment in *Drosophila* (Hall, 2000; Van Gelder, 2002) may emerge as a candidate that mediates light-induced suppression of aaNAT activity in invertebrates. Actually, the action spectrum for light-induced suppression of aaNAT activity in *T. urticae* (Suzuki et al., 2009a) is similar to the absorption spectra of *Drosophila* cryptochrome (Berndt et al., 2007). Functional analysis of these photoreceptor candidates are needed to reveal the phototransduction for aaNAT regulation in invertebrates.

## Antioxidative protection

In addition to the involvement in circadian system, MEL has been discovered to be a potent free radical scavenger and antioxidant (Reiter et al., 1994, 1997, 2000, 2007, 2009, 2010, 2013; Tan et al., 2000, 2002, 2007, 2013; Galano et al., 2011). The antioxidative properties of MEL are found in almost all living organisms (Hardeland and Poeggeler, 2003) and the capability to reduce oxidative damage is higher than the classic antioxidants such as vitamin C, vitamin E, and glutathione (Tan et al., 2013). VT-aaNAT and NV-aaNAT have been found in bacteria, unicellular chlorophyceans, fungi, and some of animals (Coon and Klein, 2006; Pavlicek et al., 2010), but not in higher plants until 2013 (Tan et al., 2012). In 2013, a NAT cDNA was cloned from GNAT cDNAs from rice and it showed high activity of *N*-acetylation of serotonin (Kang et al., 2013). The serotonin *N*-acetyltransferase (SNAT) homologs are found in cyanobacteria to higher plants (Byeon et al., 2013; Kang et al., 2013; Lei et al., 2013). Animal aaNAT is localized in mitochondria (Kerenyi et al., 1979) and plant SNAT is localized in both mitochondria and chloroplasts (Tan et al., 2013; Byeon et al., 2014). Since these organelles are the main source of free radicals generated in the courses of respiration and photosynthesis, they acquired the MEL synthetic function to reduce oxidative damages (Tan et al., 2013). It is considered that mitochondria and chloroplasts were originally transferred from bacterial species endosymbiosed with the eukaryotic host cells (Sagan, 1967); thus, the highly-conserved and widely-distributed function for MEL synthesis might be supported by these organelles even in the course of evolution. Higher MEL levels in plants than animals can also be explained by the presence of both mitochondria and chloroplasts in plant cells (Tan et al., 2013).

## Physiological and behavioral functions regulated by MEL and aaNAT in the phylum arthropoda

### Insects

**Figure 4:**
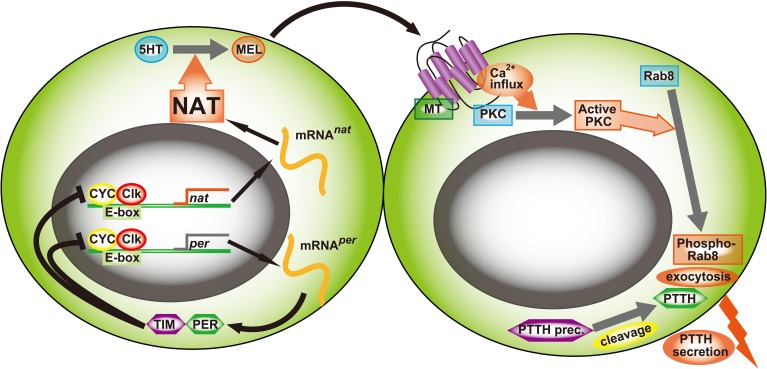
**An illustration of the coupling between the circadian neurons and PTTH-secreting neurons that control photoperiodism in *A. pernyi***. The site of coupling is at aaNAT transcription. aaNAT gene, a clock controlled gene, has several E-box enhancer elements where Cyc/Clk heterodimer binds to stimulate nat transcription. Up-regulation of this process leads to NAT translation that changes a balance between 5HT and MEL in favor of the latter. MEL stimulates PTTH synthesis/secretion in *A. pernyi* as Richter et al. (2000) and Huybrechts et al. (2005) demonstrated in *P. americana* (PTTH) and *Locusta migratoria* (AKH-related peptide precursor peptides, neuroparsins), respectively. All instrumentation for MEL synthesis is co-localized in circadian neurons in the dorsolateral protocerebrum. They juxtapose the PTTH neurons that have both MT (Mohamed et al., 2014) and two 5HTRs (Wang et al., 2013). The MEL receptor seems to be the type two-like because anti-hMT2 antibody reacted to these neurons and luzindol was effective to block MEL action. Since the same cells were reactive to anti-Rab8, PKC and PTTH antisera in *P. americana* (Hiragaki et al., 2009), the most likely process of stimulation by MEL binding is coupling to the Gq/PLC/Ca^2+^ pathway as in mouse photoreceptor cells (Baba et al., 2013) then PKC phosphorylates Rab8 required for exocytotic release of PTTH.

NAT has first been studied in conjunction with sclerotization in insects as described before (Karlson et al., 1962; Sekeris and Karlson, 1962), since the coupling between chitin and arthropodin is made using monoamine quinones that are provided by *N*-acetylated amines such as *N*-acetyl dopamine. This is one of the two major metabolic pathways of sclerotization in insects (Hopkins and Krames, 1992). Sclerotization is a unique need for arthropod where other catecolamines and phenolamines such as norepinephrine, tyramine, and octopamine are used as substrates. A periodical flood of these monoamines in molting cycle must have affected the usage of these amines as neurotransmitters. In vertebrates, these amines are metabolized by MOAs but these enzymes have limited activity in insects (Dewhurst et al., 1972). NAT took over this role. Arylalkylamines are not restricted in the nervous system unlike in vertebrates where aaNAT's major role is a MEL synthesis. We have investigated the possibility that iaaNAT also plays a similar role in arthropods. First we asked whether MEL content fluctuates in a circadian fashion. It did indeed in the brain-SOG in *P. americana* (Bembenek et al., 2005a) and *A. pernyi* (Mohamed et al., 2014). MEL in drinking water synchronized both entrained and free-running rhythms in locomotor activity of *A. domestica* (Yamano et al., 2001). NAT activity and NAT transcript showed a circadian fluctuation in the cockroach CNS. Circadian fluctuations in NAT activity, mRNA content, and MEL have been documented also in many other species (Itoh et al., 1995a,b, 1997; Itoh and Sumi, 1998a,b). We then investigated the neuronal localization of dNAT-ir in the brain-SOG complex of *B. mori* (Sehadová et al., 2004). The NAT-ir was colocalized in *Pa*Per-, dCyc-, *Bm*DBT- and dCRY-ir neurons of dorsolateral protocerebrum and SOG (Sehadová et al., 2004). The results showed that NAT is produced in some of the circadian neurons. In *P. americana* the colocalization of NAT-ir with Per- and DBT-ir was observed in the protocerebral neurons but NAT-ir was missing in the circadian neurons in the optic lobe (unpublished data). In *A. pernyi*, NAT-, HIOMT- and MEL-ir were found in Per-, Cyc-, and CLK-ir neurons of the dorsolateral protocerebrum (Mohamed et al., 2014). These clock neurons juxtapose PTTH-ir neurons that express the MEL receptor (MT)- and two serotonin receptors (5HTRs)-ir (Wang et al., 2013). The regulation of PTTH release depends on several neurotransmitters such as 5HT, glutamate, and acetylcholine in *B. mori* (Aizono et al., 1997) in *in vitro* cultures containing the brain and prothoracic glands. In *P. americana*, 5HT inhibited PTTH release while MEL stimulated its release (Richter et al., 2000). However, how these neurotransmitters themselves are controlled remained unanswered, although the gated release of PTTH has been documented in many species (ex., Vafopoulou and Steele, 1996). We have identified the critical conjunction between circadian clock and gating device. We cloned cDNA encoding aaNAT from *A. pernyi* (Tsugehara et al., 2007, 2013). This sequence had multiple E-boxes that are CLK/CYC binding enhancer elements. DsRNA^cyc^ and dsRNA^clk^ both knocked out NAT transcription and moreover dsRNA^per^ enhanced NAT transcription which confers that a simple *Drosophila* type circadian system having a negative feed-back loop operates. When dsRNA^iaaNAT^ was injected into diapause pupae under a long day, the long day failed to break diapause (Mohamed et al., 2014). Photoperiodism was indeed dysfunctioned. It was concluded that the circadian system controls NAT transcription that leads to a circadian fluctuation in NAT activity which regulates a relative balance between 5HT and MEL both daily and seasonally. One of the 5HTRs expressed in PTTH neurons locks the release of PTTH, which leads to the induction and maintenance of diapause (Hiragaki et al., 2008). The level of NAT activity depends on the circadian system according to seasonal changes in day-length (Wang et al., 2013; Mohamed et al., 2014). The NAT activity level is up-regulated by long day conditions whereas down-regulated by short day conditions. DsRNA^5HTRB^ led to diapause termination even under short day (Wang et al., 2013). Therefore, photoperiodism depends on a dual control mechanism, diapause induction/ maintenance vs. diapause termination/ avoidance as a Yin/Yang system via transcriptional regulation of *NAT* as a clock controlled gene (ccg) as depicted in Figure 4.

### Crustaceans

A wide range of physiological events are regulated by 5HT/MEL metabolic pathways in Decapoda including neuritogenesis and neuroprotective effects on X-organ neurosecretory neurons (Cary et al., 2012), induction of precocious molting in the crab, *Oziotelphusa senex senex* (Sainath and Reddy, 2010), and modification and amplitude of photoreceptor potential and ERG in the crayfish, *Procambarus clarkia* (Solis-Chagoyan et al., 2008). In the esterine crab, *Neohelice granulate*, MEL increased the oxygen consumption, glutamate cysteine ligase activity and glutathione contents, while decreasing MEL content in the antioxidant defense system of locomotor muscles via mitochondrial mechanisms (Geihs et al., 2010). Correlations between hemolymph MEL and day/night and depth differences have been observed in the Norwegian lobster, *Nephrops norvegicus* (Aguzzi et al., 2009). MEL rhythm has been observed in the optic lobes of the crab, *N. grabulata* (Maciel et al., 2008) and NAT from the giant tiger shrimp has been characterized (Withyachumnarnkul et al., 2007); K_m_ = 22 μM, V_max_ = 100 pmole/h/μg protein).

### Mites

Ever since life first appeared on the earth, it has been periodically exposed to solar radiation containing UV radiation which directly damages DNA and produces harmful reactive oxygen species (ROS) including free radicals and other reactive oxygen intermediates such as superoxide radicals, hydroxyl radicals, hydrogen peroxide and singlet oxygen as a result of electron or energy transfer to oxygen, and these cause lipid peroxidation and DNA damage (Jurkiewicz and Buettner, 1994; Shindo et al., 1994; He and Häder, 2002).

At the earliest times when the protective ozone layer was insufficient, organisms would be required to avoid and repair the damages caused by intense UV radiation, particularly UV-C (100–280 nm) and UV-B (280–315 nm). Even with the developed ozone layer today, a part of UV-B can penetrate through it and most of organisms still need the defenses against UV-B damages. Actually, most of spider mites died or escaped from UV-B at a dose equivalent to field levels (Suzuki et al., 2009b, 2013, 2014). Therefore, in addition to free radicals associated with own respiration and photosynthesis, organisms require to equip a protection system against oxidative damages caused by UV-B-induced ROS. MEL may also function as a scavenger of UV-B-induced ROS. Actually, the MEL concentration in the roots of the medicinal plant *Glycyrrhiza uralensis* is elevated by UV-B irradiation (Afreen et al., 2006). In *T. urticae*, the aaNAT activity and MEL content were suppressed at a low intensity of UV-B as well as visible light, but elevated only at a high intensity of UV-B (Suzuki et al., 2008). Although the antioxidant cascade of MEL via electron donation has been well investigated (Tan et al., 2002; Reiter et al., 2007), the upstream mechanisms of the activation of aaNAT and SNAT by UV-B irradiation or UV-B-induced ROS still remain unclarified.

### Non-vertebrate animals other than arthropods

In the gastropod, *Helix asperrsa*, aaNAT and HIOMT activities are known but the data suggest that 5 metoxytryptophol instead of MEL is released into the general circulation (Blanc et al., 2003). In *C. elegans*, MEL exogenously applied decreased locomotor activities via MT-1 receptor because luzindol, a MT-1/2 antagonist, blocked the effect of MEL, while 4P-PDOT, a MT2 specific antagonist and Prazoin, a MT-3 antagonist had no effect (Tanaka et al., 2007).

### Fungi and higher plants

A homolog of aaNAT to vertebrate timezyme has been cloned from *Sacchromyces cerevisiae* (Ganguly et al., 2001) despite the broader substrate specificity and higher pH optimum. scaaNAT has 47% similarity to ovine aaNAT but lacks the regulatory *N*- and *C*-terminal flanking regions conserved in all the vertebrate type aaNATs. The most critical difference from vertebrate type seems that scaaNAT lacks the regulatory domain that most vertebrate type enzymes have, which may be essential for photochemical transduction.

cDNA encoding SNAT has been cloned from rice, though vertebrate type aaNAT is absent from the plant genome (Kang et al., 2013). GCN5-related enzyme (GNAT) is identified as SNAT in plants. K_m_ and V_max_ were 385 μM and 282 pmol/min/mg protein, respectively, while peak activity occurred at pH 8.8. Substrate inhibition was observed. The rate limiting process is at *N*-acetylserotonin methyltransferase but not at SNAT because the latter is constitutively expressed. In rice endogenous MEL peak is observed at night as in vertebrates (Park et al., 2013a). MEL-rich transgenic rice plants became resistant to herbicide-induced oxidative stress (Park et al., 2013b). Similarly, MEL promotes water-stress tolerance, lateral root formation, and seed germination in cucumber (Zhang et al., 2013). MEL may be involved in preservation of chlorophyll, promotion of photosynthesis, and stimulation of root development (Tan et al., 2012).

## aaNAT as a potential target for pest management

**Figure 5:**
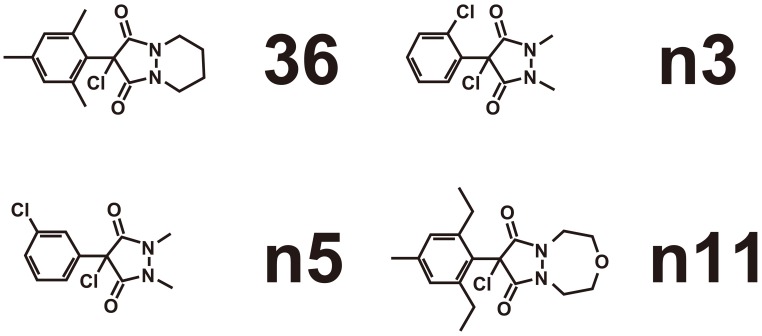
**Screening using chemical library on the expressed *ap*aaNAT by Baculovirus expression system revealed several compounds that had inhibitor activity**. These were effective also on *bm*aaNAT expressed in the same way (Tsugehara et al., 2007, 2013).

Since aaNAT regulates a wide variety of functions and the enzyme in insects are differentiated from human counterparts, disturbance of this system should provide the ideal measures for pest management. We therefore cloned cDNAs encoding *Bombyx* and *Antheraea* NATs, and expressed by means of the *Baculovirus* rapid expression system (Tsugehara et al., 2007, 2013). Using a reconstructed enzyme, a compound library was screened and compounds of high inhibitory activity were identified (Figure 5). The structure of these inhibitors is not related to any known inhibitors of sheep aaNAT. These compounds were already screened as antifungal agents. Related compounds are registered as herbicidal agents. The mode of action is known as acetyl CoA carboxylase inhibition. These compounds are reported and registered as miticidal, which indicates that these compounds penetrate the cuticle. The more systematic approach should gain more potent and compounds with species specificity in the future.

As has been shown above, the two spotted spider mite avoids UV-blue light. Therefore, UV-blue light can be employed to repel the mite from the attack site. The illumination of the mite to short-wavelength light not only affects photoperiodic determination and suppresses their settlement but affects mortality and the rate of oviposition. NAT synthesizing pathway is sensitive to this range of light and this enzyme induces MEL, a scavenger of reactive oxygen species (ROS), to rescue the mite from ROS attack. Disruption of this enzyme system should bring (1) disruption of their photoperiodism, (2) disruption of settlement, (3) reduction in oviposition and (4) reduction of defense against ROS (5) inhibition of detoxification of monoamine neurotransmitters, (6) disruption of cuticle synthesis and suppression of reproductive maturation. Integrated approach wisely combining UV exposure and inhibitor of NAT may provide most effective measures for control phytophagous mites.

## Conclusions

NAT superfamily of enzyme (GNAT) regulates a diverse physiological, morphological and behavioral functions and evolved distinct lineages of substrate specificities including arylamines, arylalkylamines, hydrazines, histone etc. even in organisms outside animal kingdom. Phylogenetic analysis of sequence similarity depicted each phylum has discrete clade but cladistic continuity is often disrupted. For example iaaNAT is not restricted to protostomial lineage but occurs in deuterostomia. Possible explanation could be horizontal transfer or opportunistic selection of a particular type of enzyme depending on phylogenetic constraint such as derivation of eyes, exoskeleton vs. endoskeleton, UV stress etc. Good correspondence of each phylum to a particular type of NAT, instead of complete randomness, favors the latter interpretation.

iaaNAT as a timezyme has been demonstrated in the photoperiodic regulation of pupal diapause in *A. pernyi* (Wang et al., 2013; Mohamed et al., 2014). These findings demonstrate the importance of indolamine metabolic pathways in understanding insect physiology and behavior. This in turn suggests that chemical manupulation of this system is an effective maneuver for integrated pest management as shown in Tsugehara et al. (2013).

### Conflict of interest statement

The authors declare that the research was conducted in the absence of any commercial or financial relationships that could be construed as a potential conflict of interest.

## Abbreviations

aaNAT: arylalkylamine *N*-acetyltransferase
iaaNAT: insect aaNAT
5HT: serotonin
MEL: melatonin
DAT: dopamine acetyltransferase
MAO: monoamine oxidase
aNAT: acidic aaNAT
bNAT: basic aaNAT
SOG: subesophageal ganglion
SCN: suprachiasmatic nucleus
CPM: circadian pacemaker
PT: pars tuberalis
MT: MEL receptor
GNAT: GCN5-related *N*-acetyltransferase
VT-aaNAT: vertebrate aaNAT
NV-aaNAT: non-vertebrate aaNAT
PTTH: prothoracicotropic hormone
GNA: glucosamine 6-phosphate *N*-acetyltransferases
HAT: histone acetyltransferase
RGC: retinal ganglion cell
ipRGC: intrinsically photosensitive RGC
SNAT: serotonin *N*-acetyltransferas.
