# Supplementary material for: Structures and functions of insect arylalkylamine N-acetyltransferase (iaaNAT); a key enzyme for physiological and behavioral switch in arthropods
Source: Front Physiol. 2015 Apr 13;6:113. doi: 10.3389/fphys.2015.00113 (PMC4394704; doi:10.3389/fphys.2015.00113)
Supplement: Supplementary file 1 [file DataSheet1.PDF]

Supporting Information

Public genome databases used in this study

Public genome databases of *Drosophila melanogaster* (*Dm*); *Apis mellifera* (*Am*; European honey bee), *Bombyx mori* (*Bm*; domesticated silkmoth); *Tribolium castaneum* (*Tc*, red flour beetle); *Acyrthosiphon pisum* (*Api*, pea aphid); *Zootermopsis nevadensis* (*Zn*, dampwood termites); *Pediculus humanus corporis* (*Phc*, body louse); *Daphnia pulex* (*Dp*); *Ixodes scapularis* (*Is*, deer tick); *Metaseiulus occidentalis* (*Mo*, western orchard predatory mite); *Tetranychus urticae* (*Tu*, two-spotted spider mite); *Caenorhabditis elegans* (*Ce*); *Ancylostoma ceylanicum* (*Ace*, eelworm); *Aplysia californica* (*Aca*); *Lottia gigantea* (*Lg*, owl limpet); *Capitella teleta* (*Ct*, polychaete); *Helobdella robusta* (*Hr*, leech); *Clonorchis sinensis* (*Cs*, flatworms); *Echinococcus granulosus* (*Eg*, flatworms); *Nematostella vectensis* (*Nv*, starlet sea anemone); *Hydra vulgaris* (*Hv*); *Amphimedon queenslandica* (*Aq*, sponges); *Trichoplax adhaerens* (*Ta*, placozoans); *Saccoglossus kowalevskii* (*Sk*, acorn worm); *Sp*, *Strongylocentrotus purpuratus* (*Sp*, purple sea urchin); *Ciona intestinalis* (*Ci*, vase tunicate); *Branchiostoma floridae* (*Bf*, Florida lancelet); *Callorhinchus milii* (*Cm*, elephant shark); *Danio rerio* (*Dr*); *Xenopus tropicalis* (*Xt*); *Gallus gallus* (*Gg*); *Python bivittatus* (*Pb*, Burmese python); and *Homo sapiens* (*Hs*) were used for this analysis.

Computational sequence analysis

The BLASTP program was used for sequence similarity searches. A cut-off for significant sequence similarity was set at an E-value $< 1.0 \times 10^{-4}$ according to data collection using *A. pisum* genomic database (Barberà et al., 2013). *Dm*DAT (GenBank Accession Number; NP_995934), *Dm*iaaNAT2 (NP_609005), *Bm*iaaNAT1 (NP_001073122), *Pa*iaaNAT (BAC87874), *A. aegypti* (*Aa*) iaaNAT5b (XP_001663019), *Cm*NV-aaNAT (XP_007901149), and *Hs*VT-aaNAT (NP_001160051) were used as queries. The CD-search tool available at NCBI was used to find the domains and motifs described for acetyltransferase family. Sequence alignments were performed with the CLUSTALW incorporated in MEGA6 (Tamura et al., 2013), which was used for phylogenetic tree building using the neighbor-joining algorithm on Poisson-corrected distances. Node support was estimated by bootstrap using 500 replicates. Divergence times (expert estimates) of phyla were obtained from the TimeTree.org website (Hedges et al., 2006).

Captions

Table S1 Details of results of BLASTP searches against RefSeq database of available entire genomic sequencings and of conserved domain (CD) search tool at National Center for Biotechnology Information. Red indicated acetyltransferases as predicted by CD-search tool or confirmed by *in vitro* experiments. Filled triangles indicate aaNAT came not from genomic database but known aaNAT to avoid taxonomy overlapping. Abbreviations; *D. melanogaster*, *Drosophila melanogaster*; *A. aegypti*, *Aedes aegypti*; *A. mellifera*, *Apis mellifera*; *B. mori*, *Bombyx mori*; *A. pernyi*, *Antheraea pernyi*; *T. castaneum*, *Tribolium castaneum*; *A. pisum*, *Acyrthosiphon pisum*; *Z. nevadensis*, *Zootermopsis nevadensis*; *P. americana*, *Periplaneta americana*; *P. h. corporis*, *Pediculus humanus corporis*; *D. pulex*, *Daphnia pulex*; *I. scapularis*, *Ixodes scapularis*; *M. occidentalis*, *Metaseiulus occidentalis*; *T. urticae*, *Tetranychus urticae*; *C. elegans*, *Caenorhabditis elegans*; *A. ceylanicum*, *Ancylostoma ceylanicum*; *A. californica*, *Aplysia californica*; *L. gigantea*, *Lottia gigantea*; *C. teleta*, *Capitella teleta*; *H. robusta*, *Helobdella robusta*; *C. sinensis*, *Clonorchis sinensis*; *E. granulosus*, *Echinococcus granulosus*; *N. vectensis*, *Nematostella vectensis*; *H. vulgaris*, *Hydra vulgaris*; *A. queenslandica*, *Amphimedon queenslandica*; *T. adhaerens*, *Trichoplax adhaerens*; *S. purpuratus*, *Strongylocentrotus purpuratus*; *S. kowalevskii*, *Saccoglossus kowalevskii*; *C. intestinalis*, *Ciona intestinalis*; *B. floridae*, *Branchiostoma floridae*; *C. milii*, *Callorhinchus milii*; *D. rerio*, *Danio rerio*; *X. tropicalis*, *Xenopus tropicalis*; *G. gallus*, *Gallus gallus*; *P. bivittatus*, *Python bivittatus*; *H. sapiens*, *Homo sapiens*; *Dm*, *D. melanogaster*; *Bm*, *B. mori*; *Pa*, *P. americana*; *Aa*, *A. aegypti*; *Cm*, *C. milii*; *Hs*, *H. sapiens*; DAT, dopamine *N*-acetyltransferase; aaNAT, arylalkylamine *N*-acetyltransferase, NV-aaNAT; non-vertebrate-type arylalkylamine *N*-acetyltransferase; NAT_SF (Accession No, cd04301), *N*-Acyltransferase superfamily; RimI (Accession No, COG0456), Acetyltransferases [General function prediction only]; COG2388 (Accession No, cl18713), Predicted acetyltransferase [General function prediction only]; Acetyltransf_1 (Accession No, pfam00583), Acetyltransferase (GNAT) family; Acetyltransf_3 (Accession No, pfam13302), Acetyltransferase (GNAT) domain; Acetyltransf_8 (Accession No, pfam13523), Acetyltransferase (GNAT) domain.
