# Supplementary material for: Structures and functions of insect arylalkylamine N-acetyltransferase (iaaNAT); a key enzyme for physiological and behavioral switch in arthropods
Source: Front Physiol. 2015 Apr 13;6:113. doi: 10.3389/fphys.2015.00113 (PMC4394704; doi:10.3389/fphys.2015.00113)
Supplement: Supplementary file 2 [file Table1.PDF]

		homology, query and E-value						
		Dm DAT1	*Dm* AANAT2	*Bm* AANAT	*Pa* AANAT	*Aa* AANAT5b	*Cm* NV-AANAT	*Hs* AANAT
	NP_995934 (DAT)	0	$7e^{-15}$	$9e^{-33}$	$3e^{-43}$	$2e^{-8}$	$>1e^{-4}$	$>1e^{-4}$
	NP_570009	$2e^{-24}$	$2e^{-14}$	$3e^{-16}$	$1e^{-35}$	$>1e^{-4}$	$>1e^{-4}$	$>1e^{-4}$
	NP_610018	$1e^{-20}$	$>1e^{-4}$	$9e^{-14}$	$2e^{-27}$	$>1e^{-4}$	$>1e^{-4}$	$>1e^{-4}$
D. melanogaster	NP_611406	$3e^{-19}$	$>1e^{-4}$	$3e^{-4}$	$2e^{-23}$	$5e^{-23}$	$>1e^{-4}$	$>1e^{-4}$
	NP_609005 (AANAT2)	$3e^{-16}$	$1e^{-158}$	$>1e^{-4}$	$2e^{-17}$	$>1e^{-4}$	$>1e^{-4}$	$>1e^{-4}$
	NP_572268	$1e^{-13}$	$1e^{-23}$	$1e^{-5}$	$4e^{-24}$	$>1e^{-4}$	$>1e^{-4}$	$>1e^{-4}$
	NP_611405	$1e^{-11}$	$2e^{-30}$	$1e^{-11}$	$5e^{-18}$	$2e^{-15}$	$>1e^{-4}$	$>1e^{-4}$
▲*A. aegypti*	XP_001663122 (AANAT2)	$2e^{-26}$	$7e^{-15}$	$1e^{-24}$	$2e^{-38}$	$6e^{-10}$	$>1e^{-4}$	$>1e^{-4}$
	XP_001663019 (AANAT5b)	$3e^{-7}$	$4e^{-5}$	$>1e^{-4}$	$>1e^{-4}$	0	$>1e^{-4}$	$>1e^{-4}$
	XP_006566050	$8e^{-42}$	$3e^{-19}$	$1e^{-39}$	$4e^{-61}$	$>1e^{-4}$	$>1e^{-4}$	$>1e^{-4}$
	XP_394768	$2e^{-21}$	$>1e^{-4}$	$2e^{-22}$	$1e^{-30}$	$6e^{-18}$	$>1e^{-4}$	$>1e^{-4}$
	XP_006564751	$1e^{-9}$	$4e^{-5}$	$>1e^{-4}$	$4e^{-12}$	$>1e^{-4}$	$>1e^{-4}$	$>1e^{-4}$
A. mellifera	XP_006560097	$3e^{-9}$	$9e^{-8}$	$>1e^{-4}$	$1e^{-22}$	$>1e^{-4}$	$>1e^{-4}$	$>1e^{-4}$
	XP_624595	$5e^{-6}$	$>1e^{-4}$	$>1e^{-4}$	$1e^{-8}$	$7e^{-5}$	$>1e^{-4}$	$>1e^{-4}$
	XP_001122379	$3e^{-5}$	$>1e^{-4}$	$>1e^{-4}$	$4e^{-10}$	$>1e^{-4}$	$>1e^{-4}$	$>1e^{-4}$
	XP_001119858	$>1e^{-4}$	$4e^{-11}$	$2e^{-22}$	$1e^{-7}$	$2e^{-33}$	$>1e^{-4}$	$>1e^{-4}$
	NP_001073122 (AANAT1)	$6e^{-30}$	$>1e^{-4}$	0	$3e^{-37}$	$>1e^{-4}$	$>1e^{-4}$	$>1e^{-4}$
	XP_004928436	$2e^{-28}$	$>1e^{-4}$	$5e^{-38}$	$8e^{-36}$	$>1e^{-4}$	$>1e^{-4}$	$>1e^{-4}$
	NP_001177771(AANAT2)	$2e^{-22}$	$1e^{-13}$	$3e^{-22}$	$7e^{-36}$	$3e^{-12}$	$>1e^{-4}$	$>1e^{-4}$
	XP_004931559	$5e^{-8}$	$>1e^{-4}$	$1e^{-10}$	$1e^{-13}$	$>1e^{-4}$	$>1e^{-4}$	$>1e^{-4}$
B. mori	XP_004926382	$5e^{-7}$	$>1e^{-4}$	$>1e^{-4}$	$3e^{-11}$	$>1e^{-4}$	$>1e^{-4}$	$>1e^{-4}$
	XP_004930734	$>1e^{-4}$	$>1e^{-4}$	$>1e^{-4}$	$9e^{-6}$	$1e^{-44}$	$>1e^{-4}$	$>1e^{-4}$
	XP_004923456	$>1e^{-4}$	$>1e^{-4}$	$>1e^{-4}$	$>1e^{-4}$	$4e^{-15}$	$>1e^{-4}$	$>1e^{-4}$
	XP_004932609	$>1e^{-4}$	$>1e^{-4}$	$>1e^{-4}$	$>1e^{-4}$	$2e^{-13}$	$>1e^{-4}$	$>1e^{-4}$
	XP_004923109	$>1e^{-4}$	$>1e^{-4}$	$>1e^{-4}$	$>1e^{-4}$	$1e^{-7}$	$>1e^{-4}$	$>1e^{-4}$
▲*A. pernyi*	ABD17803 (AANAT1)	$1e^{-28}$	$>1e^{-4}$	$1e^{-152}$	$4e^{-39}$	$>1e^{-4}$	$>1e^{-4}$	$>1e^{-4}$

TableS1-1

		Conserved Domain E-value					
		NAT_SF	RimI	COG2388	Acetyltransf_1	Acetyltransf_3	Acetyltransf_8
D. melanogaster	NP_995934 (DAT)	$3.09e^{-3}$	$3.72e^{-6}$	>0.05	>0.05	>0.05	>0.05
	NP_570009	$1.93e^{-4}$	>0.05	>0.05	>0.05	>0.05	>0.05
	NP_610018	0.02	>0.05	>0.05	>0.05	>0.05	>0.05
	NP_611406	0.01	>0.05	>0.05	>0.05	>0.05	>0.05
	NP_609005 (AANAT2)	$2.13e^{-4}$	0.01	>0.05	>0.05	>0.05	>0.05
	NP_572268	$4.08e^{-5}$	>0.05	>0.05	>0.05	>0.05	>0.05
	NP_611405	>0.05	>0.05	>0.05	>0.05	>0.05	>0.05
▲ *A. aegypti*	XP_001663122 (AANAT2)	$2.69e^{-6}$	>0.05	>0.05	>0.05	>0.05	>0.05
	XP_001663019 (AANAT5b)	$1.33e^{-3}$	$1.00e^{-4}$	>0.05	>0.05	>0.05	>0.05
A. mellifera	XP_006566050	$1.47e^{-5}$	>0.05	>0.05	>0.05	>0.05	>0.05
	XP_394768	$7.01e^{-3}$	>0.05	$3.58e^{-4}$	>0.05	>0.05	>0.05
	XP_006564751	$3.27e^{-3}$	>0.05	>0.05	>0.05	>0.05	>0.05
	XP_006560097	$1.09e^{-3}$	>0.05	>0.05	>0.05	>0.05	>0.05
	XP_624595	$3.84e^{-5}$	$4.10e^{-4}$	>0.05	>0.05	>0.05	>0.05
	XP_001122379	0.05	>0.05	>0.05	>0.05	>0.05	>0.05
	XP_001119858	$4.77e^{-4}$	>0.05	>0.05	>0.05	>0.05	>0.05
B. mori	NP_001073122 (AANAT1)	0.01	>0.05	>0.05	>0.05	>0.05	>0.05
	XP_004928436	$4.69e^{-3}$	>0.05	>0.05	>0.05	>0.05	>0.05
	NP_001177771(AANAT2)	$4.40e^{-3}$	>0.05	>0.05	>0.05	>0.05	>0.05
	XP_004931559	$1.43e^{-5}$	>0.05	>0.05	>0.05	>0.05	>0.05
	XP_004926382	>0.05	>0.05	>0.05	>0.05	>0.05	>0.05
	XP_004930734	$8.99e^{-3}$	$3.82e^{-4}$	>0.05	>0.05	>0.05	>0.05
	XP_004923456	0.04	>0.05	>0.05	>0.05	>0.05	>0.05
	XP_004932609	>0.05	>0.05	>0.05	>0.05	>0.05	>0.05
	XP_004923109	>0.05	>0.05	>0.05	>0.05	>0.05	>0.05
▲ *A. pernyi*	ABD17803 (AANAT1)	>0.05	>0.05	>0.05	>0.05	>0.05	>0.05

TableS1-2

		homology, query and E-value						
		Dm DAT1	*Dm* AANAT2	*Bm* AANAT	*Pa* AANAT	*Aa* AANAT5b	*Cm* NV-AANAT	*Hs* AANAT
T. castaneum	NP_001139379	$9e^{-50}$	$5e^{-22}$	$2e^{-37}$	$2e^{-65}$	$2e^{-8}$	$>1e^{-4}$	$>1e^{-4}$
	XP_972873	$2e^{-22}$	$7e^{-7}$	$7e^{-21}$	$1e^{-33}$	$2e^{-16}$	$>1e^{-4}$	$>1e^{-4}$
	XP_972121	$1e^{-11}$	$>1e^{-4}$	$3e^{-8}$	$>1e^{-4}$	$>1e^{-4}$	$>1e^{-4}$	$>1e^{-4}$
	XP_973841	$1e^{-7}$	$8e^{-5}$	$3e^{-16}$	$2e^{-22}$	$>1e^{-4}$	$>1e^{-4}$	$>1e^{-4}$
	XP_001812133	$>1e^{-4}$	$>1e^{-4}$	$>1e^{-4}$	$>1e^{-4}$	$3e^{-10}$	$>1e^{-4}$	$>1e^{-4}$
A. pisum	NP_001155479 (AANAT1)	$3e^{-40}$	$6e^{-15}$	$1e^{-34}$	$6e^{-57}$	$>1e^{-4}$	$>1e^{-4}$	$>1e^{-4}$
	NP_001155393 (AANAT2)	$3e^{-34}$	$1e^{-22}$	$6e^{-24}$	$1e^{-45}$	$2e^{-6}$	$>1e^{-4}$	$>1e^{-4}$
	NP_001267493 (AANAT3)	$4e^{-30}$	$1e^{-12}$	$5e^{-27}$	$1e^{-46}$	$>1e^{-4}$	$>1e^{-4}$	$>1e^{-4}$
	NP_001191994 (AANAT4)	$8e^{-28}$	$3e^{-11}$	$7e^{-23}$	$2e^{-45}$	$3e^{-6}$	$>1e^{-4}$	$>1e^{-4}$
	XP_003246857	$1e^{-12}$	$>1e^{-4}$	$2e^{-13}$	$4e^{-24}$	$>1e^{-4}$	$>1e^{-4}$	$>1e^{-4}$
	XP_001949957	$3e^{-6}$	$1e^{-4}$	$>1e^{-4}$	$2e^{-8}$	$>1e^{-4}$	$>1e^{-4}$	$>1e^{-4}$
	XP_003245537	$>1e^{-4}$	$>1e^{-4}$	$>1e^{-4}$	$>1e^{-4}$	$1e^{-11}$	$>1e^{-4}$	$>1e^{-4}$
Z. nevadensis	KDR23746	$1e^{-43}$	$5e^{-21}$	$2e^{-44}$	$3e^{-141}$	$2e^{-6}$	$>1e^{-4}$	$>1e^{-4}$
	KDR23147	$4e^{-36}$	$3e^{-18}$	$2e^{-41}$	$4e^{-70}$	$>1e^{-4}$	$>1e^{-4}$	$>1e^{-4}$
	KDR13106	$5e^{-28}$	$8e^{-14}$	$3e^{-21}$	$1e^{-40}$	$1e^{-15}$	$>1e^{-4}$	$>1e^{-4}$
	KDR14224	$1e^{-18}$	$2e^{-22}$	$8e^{-15}$	$2e^{-37}$	$>1e^{-4}$	$>1e^{-4}$	$>1e^{-4}$
	KDR22563	$2e^{-8}$	$6e^{-7}$	$2e^{-6}$	$2e^{-13}$	$3e^{-7}$	$>1e^{-4}$	$>1e^{-4}$
	KDR21523	$>1e^{-4}$	$>1e^{-4}$	$>1e^{-4}$	$1e^{-6}$	$>1e^{-4}$	$>1e^{-4}$	$>1e^{-4}$
	KDR21524	$>1e^{-4}$	$>1e^{-4}$	$>1e^{-4}$	$>1e^{-4}$	$4e^{-8}$	$>1e^{-4}$	$>1e^{-4}$
▲ *P. americana*	BAC87874	$1e^{-45}$	$5e^{-20}$	$3e^{-44}$	0	$2e^{-6}$	$>1e^{-4}$	$>1e^{-4}$
P. h. corporis	XP_002427171	$1e^{-35}$	$1e^{-12}$	$7e^{-30}$	$8e^{-54}$	$>1e^{-4}$	$>1e^{-4}$	$>1e^{-4}$
	XP_002426789	$2e^{-17}$	$1e^{-6}$	$1e^{-7}$	$8e^{-25}$	$>1e^{-4}$	$>1e^{-4}$	$>1e^{-4}$
	XP_002426957	$9e^{-12}$	$9e^{-10}$	$4e^{-9}$	$4e^{-21}$	$6e^{-9}$	$>1e^{-4}$	$>1e^{-4}$
	XP_002432214	$>1e^{-4}$	$3e^{-10}$	$2e^{-6}$	$1e^{-7}$	$8e^{-30}$	$>1e^{-4}$	$>1e^{-4}$
	XP_002426958	$>1e^{-4}$	$>1e^{-4}$	$3e^{-5}$	$4e^{-5}$	$>1e^{-4}$	$>1e^{-4}$	$>1e^{-4}$
	XP_002430761	$>1e^{-4}$	$>1e^{-4}$	$>1e^{-4}$	$2e^{-5}$	$>1e^{-4}$	$>1e^{-4}$	$>1e^{-4}$

		Conserved Domain E-value					
		NAT_SF	RimI	COG2388	Acetyltransf_1	Acetyltransf_3	Acetyltransf_8
T. castaneum	NP_001139379	$3.69e^{-5}$	>0.05	>0.05	>0.05	>0.05	>0.05
	XP_972873	$2.33e^{-4}$	>0.05	>0.05	>0.05	>0.05	>0.05
	XP_972121	>0.05	>0.05	>0.05	>0.05	>0.05	>0.05
	XP_973841	0.01	>0.05	>0.05	>0.05	>0.05	>0.05
	XP_001812133	>0.05	>0.05	>0.05	>0.05	>0.05	>0.05
A. pisum	NP_001155479 (AANAT1)	$1.55e^{-6}$	>0.05	>0.05	>0.05	>0.05	>0.05
	NP_001155393 (AANAT2)	$1.50e^{-5}$	$8.23e^{-3}$	>0.05	>0.05	>0.05	>0.05
	NP_001267493 (AANAT3)	$3.76e^{-5}$	>0.05	>0.05	>0.05	>0.05	>0.05
	NP_001191994 (AANAT4)	$1.49e^{-4}$	>0.05	>0.05	>0.05	>0.05	>0.05
	XP_003246857	>0.05	>0.05	>0.05	>0.05	>0.05	>0.05
	XP_001949957	>0.05	>0.05	>0.05	>0.05	>0.05	>0.05
	XP_003245537	$1.95e^{-5}$	>0.05	>0.05	>0.05	$4.59e^{-3}$	>0.05
Z. nevadensis	KDR23746	$7.96e^{-6}$	>0.05	>0.05	>0.05	>0.05	>0.05
	KDR23147	$2.20e^{-6}$	>0.05	>0.05	>0.05	>0.05	>0.05
	KDR13106	0.03	>0.05	>0.05	>0.05	>0.05	>0.05
	KDR14224	$2.06e^{-6}$	>0.05	>0.05	>0.05	>0.05	>0.05
	KDR22563	>0.05	>0.05	>0.05	>0.05	>0.05	>0.05
	KDR21523	>0.05	>0.05	>0.05	>0.05	>0.05	>0.05
	KDR21524	>0.05	>0.05	>0.05	>0.05	>0.05	>0.05
▲ *P. americana*	BAC87874	$2.10e^{-6}$	>0.05	>0.05	>0.05	>0.05	>0.05
P. h. corporis	XP_002427171	$3.61e^{-5}$	>0.05	>0.05	>0.05	>0.05	>0.05
	XP_002426789	$1.21e^{-9}$	>0.05	>0.05	>0.05	>0.05	>0.05
	XP_002426957	$1.27e^{-4}$	>0.05	>0.05	>0.05	>0.05	>0.05
	XP_002432214	>0.05	>0.05	>0.05	>0.05	>0.05	>0.05
	XP_002426958	>0.05	>0.05	>0.05	>0.05	>0.05	>0.05
	XP_002430761	$7.74e^{-3}$	>0.05	>0.05	>0.05	>0.05	>0.05

TableS1-4

		homology, query and E-value						
		Dm DAT1	*Dm* AANAT2	*Bm* AANAT	*Pa* AANAT	*Aa* AANAT5b	*Cm* NV-AANAT	*Hs* AANAT
D. pulex	EFX76241	$2e^{-14}$	$8e^{-8}$	$4e^{-8}$	$5e^{-17}$	$>1e^{-4}$	$>1e^{-4}$	$>1e^{-4}$
	EFX76660	$6e^{-13}$	$1e^{-5}$	$9e^{-9}$	$6e^{-19}$	$>1e^{-4}$	$>1e^{-4}$	$>1e^{-4}$
	EFX79773	$1e^{-12}$	$>1e^{-4}$	$1e^{-4}$	$2e^{-10}$	$>1e^{-4}$	$>1e^{-4}$	$>1e^{-4}$
	EFX79774	$2e^{-11}$	$>1e^{-4}$	$>1e^{-4}$	$9e^{-10}$	$>1e^{-4}$	$>1e^{-4}$	$>1e^{-4}$
	EFX76242	$2e^{-9}$	$9e^{-5}$	$5e^{-5}$	$3e^{-7}$	$5e^{-5}$	$>1e^{-4}$	$>1e^{-4}$
	EFX71659	$3e^{-7}$	$>1e^{-4}$	$>1e^{-4}$	$>1e^{-4}$	$>1e^{-4}$	$>1e^{-4}$	$>1e^{-4}$
	EFX72905	$2e^{-6}$	$>1e^{-4}$	$1e^{-4}$	$1e^{-6}$	$>1e^{-4}$	$>1e^{-4}$	$>1e^{-4}$
	EFX74382	$2e^{-6}$	$2e^{-6}$	$6e^{-5}$	$1e^{-5}$	$1e^{-6}$	$>1e^{-4}$	$>1e^{-4}$
	EFX74384	$8e^{-6}$	$2e^{-7}$	$>1e^{-4}$	$2e^{-13}$	$>1e^{-4}$	$>1e^{-4}$	$>1e^{-4}$
	EFX74383	$2e^{-5}$	$>1e^{-4}$	$>1e^{-4}$	$1e^{-6}$	$>1e^{-4}$	$>1e^{-4}$	$>1e^{-4}$
	EFX72904	$>1e^{-4}$	$>1e^{-4}$	$>1e^{-4}$	$9e^{-6}$	$>1e^{-4}$	$>1e^{-4}$	$>1e^{-4}$
I. scapularis	n/a	-	-	-	-	-	-	-
M. occidentalis	n/a	-	-	-	-	-	-	-
T. urticae	n/a	-	-	-	-	-	-	-
C. elegans	NP_502069	$8e^{-6}$	$>1e^{-4}$	$>1e^{-4}$	$>1e^{-4}$	$>1e^{-4}$	$>1e^{-4}$	$>1e^{-4}$
A. ceylanicum	EYB81954	$6e^{-8}$	$>1e^{-4}$	$>1e^{-4}$	$5e^{-6}$	$1e^{-7}$	$>1e^{-4}$	$>1e^{-4}$
	EYC12753	$7e^{-7}$	$>1e^{-4}$	$>1e^{-4}$	$1e^{-6}$	$>1e^{-4}$	$>1e^{-4}$	$>1e^{-4}$
	EYC31446	$>1e^{-4}$	$>1e^{-4}$	$9e^{-6}$	$>1e^{-4}$	$>1e^{-4}$	$>1e^{-4}$	$>1e^{-4}$
	EYC37959	$>1e^{-4}$	$>1e^{-4}$	$2e^{-5}$	$>1e^{-4}$	$>1e^{-4}$	$>1e^{-4}$	$>1e^{-4}$
	EYC12751	$>1e^{-4}$	$>1e^{-4}$	$>1e^{-4}$	$8e^{-5}$	$>1e^{-4}$	$>1e^{-4}$	$>1e^{-4}$
A. californica	n/a	-	-	-	-	-	-	-
L. gigantea	n/a	-	-	-	-	-	-	-

		Conserved Domain E-value					
		NAT_SF	RimI	COG2388	Acetyltransf_1	Acetyltransf_3	Acetyltransf_8
D. pulex	EFX76241	$5.51e^{-3}$	>0.05	>0.05	>0.05	>0.05	>0.05
	EFX76660	0.01	>0.05	>0.05	>0.05	>0.05	>0.05
	EFX79773	>0.05	>0.05	>0.05	>0.05	>0.05	>0.05
	EFX79774	>0.05	>0.05	>0.05	>0.05	>0.05	>0.05
	EFX76242	$2.17e^{-3}$	>0.05	>0.05	>0.05	>0.05	>0.05
	EFX71659	$5.84e^{-5}$	$5.75e^{-7}$	>0.05	>0.05	>0.05	>0.05
	EFX72905	>0.05	>0.05	>0.05	>0.05	>0.05	>0.05
	EFX74382	0.01	>0.05	>0.05	>0.05	>0.05	>0.05
	EFX74384	$6.69e^{-3}$	$7.84e^{-4}$	>0.05	>0.05	>0.05	>0.05
	EFX74383	$4.37e^{-3}$	>0.05	>0.05	>0.05	>0.05	>0.05
	EFX72904	>0.05	>0.05	>0.05	>0.05	>0.05	>0.05
I. scapularis	n/a	-	-	-	-	-	-
M. occidentalis	n/a	-	-	-	-	-	-
T. urticae	n/a	-	-	-	-	-	-
C. elegans	NP_502069	0.02	>0.05	>0.05	>0.05	>0.05	>0.05
A. ceylanicum	EYB81954	$3.67e^{-3}$	>0.05	>0.05	>0.05	>0.05	>0.05
	EYC12753	$1.32e^{-6}$	$7.47e^{-6}$	>0.05	>0.05	>0.05	>0.05
	EYC31446	$4.62e^{-3}$	>0.05	>0.05	>0.05	>0.05	>0.05
	EYC37959	>0.05	>0.05	>0.05	>0.05	0.01	>0.05
	EYC12751	$5.73e^{-5}$	$7.33e^{-3}$	>0.05	>0.05	>0.05	>0.05
A. californica	n/a	-	-	-	-	-	-
L. gigantea	n/a	-	-	-	-	-	-

		homology, query and E-value						
		Dm DAT1	*Dm* AANAT2	*Bm* AANAT	*Pa* AANAT	*Aa* AANAT5b	*Cm* NV-AANAT	*Hs* AANAT
C. teleta	ELU18274	>1e $^{-4}$	>1e $^{-4}$	>1e $^{-4}$	>1e $^{-4}$	>1e $^{-4}$	3e $^{-38}$	4e $^{-14}$
	ELU00181	>1e $^{-4}$	>1e $^{-4}$	>1e $^{-4}$	>1e $^{-4}$	>1e $^{-4}$	5e $^{-27}$	3e $^{-7}$
H. robusta	n/a	-	-	-	-	-	-	-
C. sinensis	n/a	-	-	-	-	-	-	-
E. granulosus	n/a	-	-	-	-	-	-	-
T. adhaerens	XP_002113076	>1e $^{-4}$	>1e $^{-4}$	>1e $^{-4}$	>1e $^{-4}$	>1e $^{-4}$	2e $^{-40}$	2e $^{-13}$
	XP_002113074	>1e $^{-4}$	>1e $^{-4}$	>1e $^{-4}$	>1e $^{-4}$	>1e $^{-4}$	9e $^{-32}$	4e $^{-10}$
	XP_002118507	>1e $^{-4}$	>1e $^{-4}$	>1e $^{-4}$	>1e $^{-4}$	>1e $^{-4}$	1e $^{-24}$	>1e $^{-4}$
	XP_002113075	>1e $^{-4}$	>1e $^{-4}$	>1e $^{-4}$	>1e $^{-4}$	>1e $^{-4}$	1e $^{-18}$	4e $^{-5}$
H. vulgaris	n/a	-	-	-	-	-	-	-
N. vectensis	n/a	-	-	-	-	-	-	-
A. queenslandica	n/a	-	-	-	-	-	-	-
S. purpuratus	n/a	-	-	-	-	-	-	-
S. kowalevskii	XP_006819946	4e $^{-8}$	>1e $^{-4}$	>1e $^{-4}$	1e $^{-5}$	3e $^{-9}$	>1e $^{-4}$	>1e $^{-4}$
	XP_006812766	7e $^{-8}$	8e $^{-7}$	1e $^{-4}$	1e $^{-7}$	>1e $^{-4}$	>1e $^{-4}$	>1e $^{-4}$
	XP_006812769	2e $^{-7}$	>1e $^{-4}$	>1e $^{-4}$	2e $^{-6}$	>1e $^{-4}$	>1e $^{-4}$	>1e $^{-4}$
	XP_006825460	>1e $^{-4}$	>1e $^{-4}$	>1e $^{-4}$	3e $^{-6}$	>1e $^{-4}$	>1e $^{-4}$	>1e $^{-4}$

		Conserved Domain E-value					
		NAT_SF	RimI	COG2388	Acetyltransf_1	Acetyltransf_3	Acetyltransf_8
C. teleta	ELU18274	$2.37e^{-4}$	$1.33e^{-5}$	>0.05	>0.05	>0.05	>0.05
	ELU00181	$1.53e^{-3}$	>0.05	>0.05	>0.05	>0.05	>0.05
H. robusta	n/a	-	-	-	-	-	-
C. sinensis	n/a	-	-	-	-	-	-
E. granulosus	n/a	-	-	-	-	-	-
T. adhaerens	XP_002113076	$1.48e^{-8}$	$2.08e^{-8}$	>0.05	>0.05	>0.05	>0.05
	XP_002113074	$1.58e^{-7}$	>0.05	>0.05	>0.05	>0.05	>0.05
	XP_002118507	$1.89e^{-7}$	>0.05	>0.05	>0.05	>0.05	>0.05
	XP_002113075	0.02	>0.05	>0.05	>0.05	>0.05	>0.05
H. vulgaris	n/a	-	-	-	-	-	-
N. vectensis	n/a	-	-	-	-	-	-
A. queenslandica	n/a	-	-	-	-	-	-
S. purpuratus	n/a	-	-	-	-	-	-
S. kowalevskii	XP_006819946	$7.27e^{-4}$	>0.05	>0.05	>0.05	>0.05	>0.05
	XP_006812766	$5.21e^{-4}$	>0.05	>0.05	>0.05	>0.05	>0.05
	XP_006812769	$6.54e^{-6}$	$4.09e^{-7}$	>0.05	>0.05	>0.05	>0.05
	XP_006825460	>0.05	>0.05	>0.05	$1.88e^{-5}$	>0.05	>0.05

		homology, query and E-value						
		Dm DAT1	*Dm* AANAT2	*Bm* AANAT	*Pa* AANAT	*Aa* AANAT5b	*Cm* NV-AANAT	*Hs* AANAT
C. intestinalis	XP_002130504	3e^{-6}	>1e^{-4}	2e^{-7}	>1e^{-4}	>1e^{-4}	>1e^{-4}	>1e^{-4}
	XP_002131416	>1e^{-4}	6e^{-6}	>1e^{-4}	1e^{-10}	>1e^{-4}	>1e^{-4}	>1e^{-4}
	XP_002126007	>1e^{-4}	1e^{-4}	>1e^{-4}	>1e^{-4}	>1e^{-4}	>1e^{-4}	>1e^{-4}
	XP_002120590	>1e^{-4}	>1e^{-4}	3e^{-6}	>1e^{-4}	>1e^{-4}	>1e^{-4}	>1e^{-4}
	XP_002123594	>1e^{-4}	>1e^{-4}	7e^{-5}	1e^{-5}	>1e^{-4}	>1e^{-4}	>1e^{-4}
	XP_004225632	>1e^{-4}	>1e^{-4}	>1e^{-4}	>1e^{-4}	>1e^{-4}	>1e^{-4}	>1e^{-4}
	XP_002125044	>1e^{-4}	>1e^{-4}	>1e^{-4}	>1e^{-4}	>1e^{-4}	>1e^{-4}	>1e^{-4}
	XP_002126193	>1e^{-4}	>1e^{-4}	>1e^{-4}	>1e^{-4}	>1e^{-4}	>1e^{-4}	>1e^{-4}
B. floridae	XP_002608038	>1e^{-4}	>1e^{-4}	>1e^{-4}	>1e^{-4}	>1e^{-4}	9e^{-56}	4e^{-12}
	XP_002608040	>1e^{-4}	>1e^{-4}	>1e^{-4}	>1e^{-4}	>1e^{-4}	7e^{-38}	2e^{-11}
	XP_002609240	>1e^{-4}	>1e^{-4}	>1e^{-4}	>1e^{-4}	>1e^{-4}	5e^{-36}	1e^{-7}
	XP_002608037	>1e^{-4}	>1e^{-4}	>1e^{-4}	>1e^{-4}	>1e^{-4}	3e^{-33}	2e^{-7}
C. milii	XP_007901149 (NV-AANAT)	>1e^{-4}	>1e^{-4}	>1e^{-4}	>1e^{-4}	>1e^{-4}	2e^{-122}	1e^{-6}
	NP_001279701 (VT-AANAT)	>1e^{-4}	>1e^{-4}	>1e^{-4}	>1e^{-4}	>1e^{-4}	2e^{-11}	8e^{-82}
D. rerio	NP_956998	>1e^{-4}	>1e^{-4}	>1e^{-4}	>1e^{-4}	>1e^{-4}	2e^{-16}	1e^{-91}
	NP_571486	>1e^{-4}	>1e^{-4}	>1e^{-4}	>1e^{-4}	>1e^{-4}	6e^{-13}	7e^{-88}
X. tropicalis	XP_002935979	>1e^{-4}	>1e^{-4}	>1e^{-4}	>1e^{-4}	>1e^{-4}	5e^{-11}	4e^{-95}
G. gallus	NP_990489	>1e^{-4}	>1e^{-4}	>1e^{-4}	>1e^{-4}	>1e^{-4}	5e^{-9}	7e^{-94}
P. bivittatus	XP_007431904	>1e^{-4}	>1e^{-4}	>1e^{-4}	>1e^{-4}	>1e^{-4}	>1e^{-4}	2e^{-90}
H. sapiens	NP_001160051	>1e^{-4}	>1e^{-4}	>1e^{-4}	>1e^{-4}	>1e^{-4}	3e^{-6}	0

		Conserved Domain E-value					
		NAT_SF	RimI	COG2388	Acetyltransf_1	Acetyltransf_3	Acetyltransf_8
C. intestinalis	XP_002130504	0.02	>0.05	>0.05	>0.05	>0.05	>0.05
	XP_002131416	>0.05	>0.05	>0.05	>0.05	>0.05	>0.05
	XP_002126007	>0.05	>0.05	>0.05	>0.05	>0.05	>0.05
	XP_002120590	>0.05	>0.05	>0.05	>0.05	>0.05	>0.05
	XP_002123594	$5.03e^{-3}$	>0.05	>0.05	>0.05	>0.05	>0.05
	XP_004225632	>0.05	>0.05	>0.05	>0.05	>0.05	>0.05
	XP_002125044	>0.05	>0.05	>0.05	>0.05	>0.05	>0.05
	XP_002126193	>0.05	>0.05	>0.05	>0.05	>0.05	>0.05
B. floridae	XP_002608038	$1.13e^{-4}$	>0.05	>0.05	>0.05	>0.05	$2.23e^{-3}$
	XP_002608040	$2.76e^{-4}$	>0.05	>0.05	>0.05	>0.05	>0.05
	XP_002609240	$2.45e^{-4}$	>0.05	>0.05	>0.05	>0.05	>0.05
	XP_002608037	$1.77e^{-7}$	>0.05	>0.05	>0.05	>0.05	>0.05
C. milii	XP_007901149 (NV-AANAT)	0.02	>0.05	>0.05	>0.05	>0.05	>0.05
	NP_001279701 (VT-AANAT)	0.02	>0.05	>0.05	>0.05	>0.05	>0.05
D. rerio	NP_956998	$1.13e^{-3}$	$8.62e^{-5}$	>0.05	>0.05	>0.05	>0.05
	NP_571486	$8.56e^{-3}$	$5.14e^{-7}$	>0.05	>0.05	>0.05	>0.05
X. tropicalis	XP_002935979	$8.34e^{-4}$	$7.52e^{-5}$	>0.05	>0.05	>0.05	>0.05
G. gallus	NP_990489	$2.12e^{-4}$	$3.33e^{-6}$	>0.05	>0.05	>0.05	>0.05
P. bivittatus	XP_007431904	$2.50e^{-3}$	$3.50e^{-5}$	>0.05	>0.05	>0.05	>0.05
H. sapiens	NP_001160051	>0.05	>0.05	>0.05	$1.62e^{-7}$	$1.62e^{-7}$	>0.05
